# Mass spectrometry imaging–based assays for aminotransferase activity reveal a broad substrate spectrum for a previously uncharacterized enzyme

**DOI:** 10.1016/j.jbc.2023.102939

**Published:** 2023-01-24

**Authors:** Markus de Raad, Kaan Koper, Kai Deng, Benjamin P. Bowen, Hiroshi A. Maeda, Trent R. Northen

**Affiliations:** 1 Environmental Genomics and Systems Biology Division, Lawrence Berkeley National Laboratory, Berkeley, California, USA; 2 Department of Botany, University of Wisconsin-Madison; Madison, Wisconsin, USA; 3 Joint BioEnergy Institute, Lawrence Berkeley National Laboratory, Emeryville, California, USA; 4 Sandia National Laboratories, Livermore, California, USA; 5 Joint Genome Institute, Lawrence Berkeley National Laboratory, Berkeley, California, USA

**Keywords:** aminotransferases, mass spectrometry, mass spectrometry imaging, high-throughput screening, enzyme screening, enzyme promiscuity, nanostructure-initiator mass spectrometry, pyridoxal-5′-phosphate–dependent enzyme, AABA, 2-aminobutyric acid, Ala, alanine, Arg, arginine, Asn, asparagine, Asp, aspartic acid, AT, Aminotransferase, *At*TAA1, *Arabidopsis thaliana* tryptophan aminotransferase of *Arabidopsis* 1, *At*TAR1, *Arabidopsis thaliana* tryptophan aminotransferase related protein 1, *At*TAT1, *Arabidopis thaliana* tyrosine aminotransferase 1, Glu, glutamic acid, His, histidine, HTS, high-throughput screening, IPA, indole-3-pyruvic acid, Leu, leucine, Met, methionine, MSI, mass spectrometry imaging, NIMS, nanostructure-initiator mass spectrometry, O-MTY, O-methyl-tyrosine, PLP, Pyridoxal 5′-phosphate, Phe, phenylalanine, Trp, tryptophan, Tyr, tyrosine, ɑ-KG, ɑ-ketoglutarate

## Abstract

Aminotransferases (ATs) catalyze pyridoxal 5′-phosphate–dependent transamination reactions between amino donor and keto acceptor substrates and play central roles in nitrogen metabolism of all organisms. ATs are involved in the biosynthesis and degradation of both proteinogenic and nonproteinogenic amino acids and also carry out a wide variety of functions in photorespiration, detoxification, and secondary metabolism. Despite the importance of ATs, their functionality is poorly understood as only a small fraction of putative ATs, predicted from DNA sequences, are associated with experimental data. Even for characterized ATs, the full spectrum of substrate specificity, among many potential substrates, has not been explored in most cases. This is largely due to the lack of suitable high-throughput assays that can screen for AT activity and specificity at scale. Here we present a new high-throughput platform for screening AT activity using bioconjugate chemistry and mass spectrometry imaging–based analysis. Detection of AT reaction products is achieved by forming an oxime linkage between the ketone groups of transaminated amino donors and a probe molecule that facilitates mass spectrometry-based analysis using nanostructure-initiator mass spectrometry or MALDI–mass spectrometry. As a proof-of-principle, we applied the newly established method and found that a previously uncharacterized *Arabidopsis thaliana* tryptophan AT-related protein 1 is a highly promiscuous enzyme that can utilize 13 amino acid donors and three keto acid acceptors. These results demonstrate that this oxime–mass spectrometry imaging AT assay enables high-throughput discovery and comprehensive characterization of AT enzymes, leading to an accurate understanding of the nitrogen metabolic network.

Aminotransferases (ATs) are a highly versatile class of pyridoxal-5′-phosphate (PLP)-dependent enzymes that transfer an amino group from an amino donor to a keto acceptor substrate *via* the ping-pong bi-bi mechanism (1, 2). ATs are ubiquitous across all kingdoms of life and are responsible for ∼2% of all the reactions that have been assigned an Enzyme Commission number (3, 4). As a result, ATs constitute the largest family of enzymes involved in nitrogen (N) metabolism (Enzyme Commission number 2.6.1.x). For example, in animals, alanine (Ala) ATs play a key role in the Cahill or glucose–Ala cycle, a process occurring in the liver tissue that involves the transamination of Ala to pyruvic acid, which is subsequently converted to glucose *via* gluconeogenesis and transported to muscle tissues for ATP production (5). In gram-negative bacteria, amino sugars found in the lipopolysaccharides present in the outer membrane are usually synthesized from keto-sugars by ATs (6). In plants, the aromatic AT ISS1 regenerates tryptophan using methionine as the amino donor, which connects the biosynthesis of two phytohormones, tryptophan-derived auxin and methionine-derived ethylene (7). Since most ATs transfer an amino group from an amino acid donor to a keto acid acceptor and generate the corresponding keto and amino acid products, AT-catalyzed reactions play pivotal roles in interconnecting different branches of metabolic pathways (8). Thus, an accurate understanding of AT functions is critical for comprehending the architecture and functionality of the N metabolic network, which is currently poorly defined as compared to the carbon metabolic network. This will be highly valuable for a broad range of applications, such as for identifying novel drug targets for cancer therapy, improving plant N use efficiency to enhance crop yield, and optimizing microbial pathways to efficiently produce renewable chemicals and biofuels (9, 10, 11).

Notably, many AT enzymes are promiscuous, exhibit broad substrate specificity, and have multiple activities which are likely critical for the plasticity of N metabolic networks (2). For example, yeast aromatic AT 8 is active with aromatic amino acids, methionine, and leucine, while the human kynurenine AT III has both AT and β-lyase activities (2, 12, 13). Despite their importance, however, the full functionalities of ATs, *i.e.*, their substrate specificity profiles, are not well defined. This suggests that there are still many AT-catalyzed reactions that could be physiologically important but are currently unknown. Thus, the large number of AT gene candidates combined with the broad substrate specificity/promiscuity contributed to a low number of fully characterized ATs (2). This large knowledge gap between AT sequence and function is further exacerbated by the lack of suitable high-throughput assays that can screen for AT activity and donor/acceptor specificity.

Conventional AT assays use the intrinsic absorbance or fluorescence of the substrates or reaction products as the readout for AT activity (14). For example, AT assays using tyrosine (Tyr) or ɑ-methylbenzylamine as amino donors measure the absorbance of the deaminated reaction products, 4-hydroxyphenylpyruvic acid and acetophenone, at 331 nm and 245 nm, respectively (15, 16). Alternatively, the reaction products can be derivatized to confer absorbance or fluorescence properties, such as the use of the chromogenic Salkowski reagent to detect the production of indole-3-pyruvic acid (IPA) in tryptophan AT assays (17). Most of these assays can screen multiple AT enzymes using a plate reader but are limited to one specific or a few AT reactions and cannot test incompatible substrate or product pairs. AT activity also can be determined indirectly by coupling the AT reaction with another, more easily detectable, reaction. For example, Ala AT activity can be coupled to the rate of NADH oxidation by lactate dehydrogenase, which forms the basis of commercial enzyme screening kits. Also, a pH-indicator assay using a pH-sensitive dye was developed where AT activity leads to a reduction in the pH through the use of a combined lactate dehydrogenase and glucose dehydrogenase system (18). Another example is the coupling of AT activity to the detection of H_2_O_2_ generated through amino acid oxidases using pyrogallol red and horseradish peroxidase (19). Similar to conventional assays, these coupled AT assays are limited to a small number of reactions with combinations of compatible reaction products. For example, lactate dehydrogenase and malate dehydrogenase need to be coupled to the reactions of ATs that act on Ala and aspartate (or pyruvate and oxaloacetate). Other assays use unnatural amino donors, where the formed keto acid analogues self-react to form chromogenic products (20). However, this limits the assay to screen only for different keto acceptors, and the AT must be able to use the unnatural amino donor. Direct analysis of AT activity using native substrates and without the need of coupled assays or derivatized reaction products can be performed using HPLC, capillary electrophoresis, circular dichroism, conductometry, or GLC coupled with mass spectroscopy and NMR (21, 22). However, these techniques are time consuming and have a limited throughput.

Nanostructure-initiator mass spectrometry (NIMS) assays are a type of mass spectrometry–based enzyme assays that overcome the above mentioned challenges on limited throughputs and specificities (23). NIMS assays are an established platform for high-throughput enzyme activity determination, in which perfluorous-tagged substrates are desorpted from a perfluorinated nanostructured silicon surface and analyzed in a mass spectrometer (23, 24, 25, 26, 27). Alternatively, MALDI or other laser desorption ionization techniques can be used to analyze perfluorous-tagged substrates. The use of perfluorous tags improves assay sensitivity by conferring favorable mass spectrometry properties to the analyte, and assays can be performed with perfluorous-tagged substrates or reaction products that can be conjugated with a perfluorous tag (28, 29). Here we report a new method to characterize AT substrate specificity based on the oxime NIMS assay (28). The method utilizes a perfluorous alkoxyamine probe that forms an oxime linkage with ketones that are present in any keto acid products after transamination of amino donors (Fig. 1*A*). Therefore, all potential AT activities can be analyzed in an unbiased manner. This probe can be added to the reaction mixtures after the transamination reactions and affords highly efficient capture of transamination products into the mass-diagnostic tag with subsequent high sensitivity analysis without separation of the reaction products (28). Arraying the reactions onto the NIMS surface using acoustic sample deposition and subsequent analysis by mass spectrometry imaging (MSI) allows for the high-throughput characterization of AT activity and substrate specificity (Fig. 1*B*) (30, 31).

**Figure 1:**
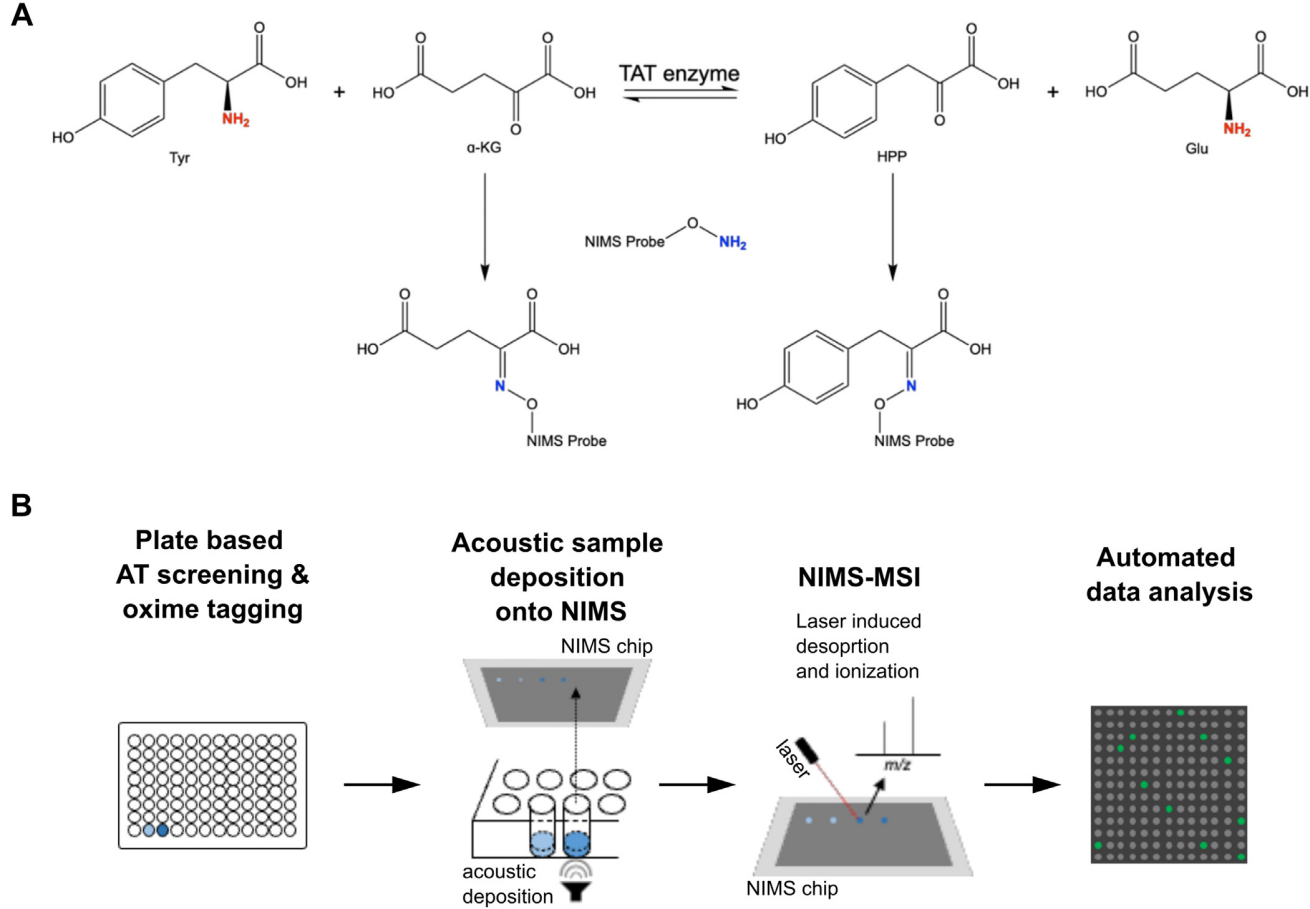
**Overview of the oxime-MSI AT assay.***A*, schematic overview of oxime tagging to detect AT activity. Tyrosine (Tyr) is, for example, transaminated by Tyr AT (TAT) using α-ketoglutarate (α-KG) as keto acceptor yielding 4-hydroxyphenylpyruvate (HPP) and glutamic acid (Glu). The aminooxy group on the oxime probe reacts with the ketone group of α-KG and HPP. The resulting covalent adduct is ready for subsequent MSI analysis. The amino donor involved in the AT reaction is shown in *red*, while the amine of the NIMS probe is in *blue*. *B*, steps in the oxime-MSI AT assay. After the enzymatic reactions and subsequent oxime tagging, samples are printed onto the NIMS surface using acoustic sample deposition. Next, MSI data is acquired and analyzed for AT activity, where positive signals are depicted as *green dots*. AT, aminotransferase; MSI, mass spectrometry imaging; NIMS, nanostructure-initiator mass spectrometry.

Here we showed that the oxime-MSI assay can be used to study substrate specificity of ATs in a high-throughput and semi-quantitative manner. We used recombinant three Tyr and tryptophan ATs from *Arabidopsis thaliana* to screen against 31 amino acid donors in combination with three keto acid acceptors. The results confirmed the previously reported broad substrate specificity of the TyrAT (15) and also revealed that the previously uncharacterized tryptophan AT, TAR1, is a highly promiscuous enzyme that can use 13 different amino donors. This new approach has great promise to rapidly improve our understanding of the substrate specificities of ATs with a broad range of combinations of amino donors and keto acceptors.

## Results

### Establishing the oxime-MSI at assay using previously characterized *At*TAT1

**Figure 2:**
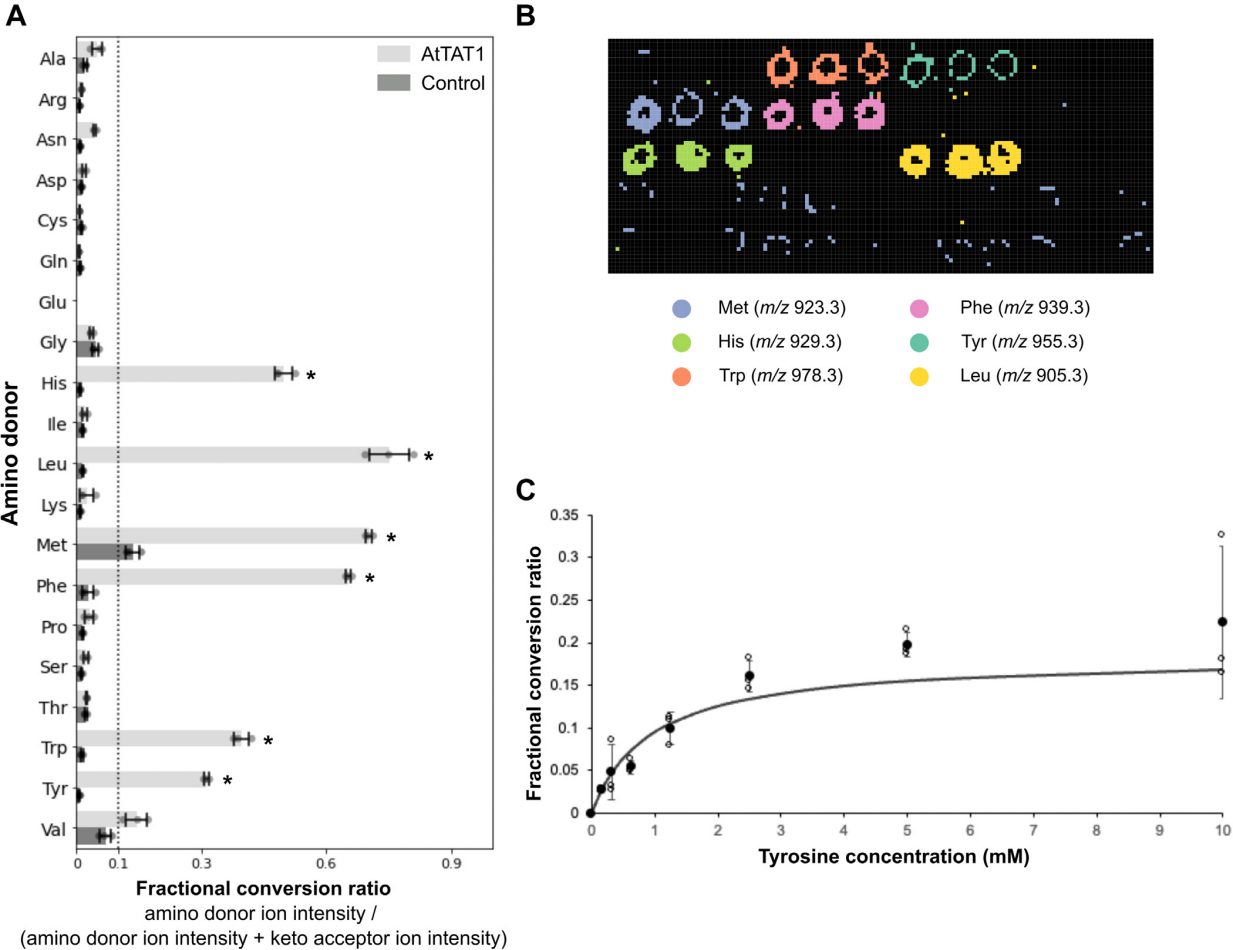
**AT amino donor specificity screening using oxime tagging and mass spectrometry analysis.***A*, aminotransferase (AT) activity of *At*TAT1 was analyzed for 20 amino acids using ɑ-KG as a keto acceptor. Bar chart shows the fractional conversion ratios for each amino donor with *At*TAT1 or the control (no amino donor) ± the standard deviations for three biological replicates. Individual biological replicates are plotted as *closed circles*. Dotted line indicates a fractional conversion ratio of 0.1. ∗ indicates a fractional conversion ratio of >0.1 and greater than 10 standard deviations compared to the control. *B*, MS image of NIMS surface with printed *At*TAT1 reactions, with the ion intensity visualization of six ions by different false colors representing the oxime probe reacted with transaminated Met, His, Trp, Phe, Tyr, and Leu. *C*, the relative reaction rate of *At*TAT1 with Tyr and ɑ-KG using various concentrations of amino donor tyrosine (0–10 mM). Production of 4-hydroxyphenylpyruvic acid was measured by oxime NIMS. Fractional conversion ratios for the different concentrations of *At*TAT1 are plotted ± the standard deviations for three biological replicates. Individual biological replicates are plotted as open circles. Solid line represents hyperbolic regression analysis with y = 0.1705 ∗ x/0.8468 + x and r^2^ = 0.67. *At*TAT1, *Arabidopis thaliana* tyrosine aminotransferase 1; Ala, alanine; Arg, arginine; Asn, asparagine; Asp, aspartic acid; Cys, cysteine; Gln, glutamine; Glu, glutamic acid; Gly, glycine; His, histidine; Ile, isoleucine; Leu, leucine; Lys, lysine; Met, methionine; NIMS, nanostructure-initiator mass spectrometry; Phe, phenylalanine; Pro, proline; Ser, serine; Thr, threonine; Trp, tryptophan; Val, valine; ɑ-KG, ɑ-ketoglutarate.

Our work is motivated by the need for a scalable methodology that would enable the high-throughput profiling of AT amino donor and keto acceptor specificities. To this end, we incorporated oxime tagging and subsequent MSI analysis using NIMS (28). For a proof-of-principle, we initially screened the detectable activity of the previously characterized *A. thaliana* Tyr AT 1 (*At*TAT1) against all 20 proteinogenic amino acids and used ɑ-ketoglutarate (ɑ-KG) as keto acceptor. *At*TAT1 was chosen because its substrate specificity has been extensively studied for each substrate one-by-one using a number of established methods (*e.g.*, spectrometric and HPLC-based assays) (15). Here we determine the conversion of these amino donors into their corresponding keto acids, which can be tagged by the NIMS probe (Fig. 1). Activity was identified as samples having a fractional conversion ratio of >0.1 and a ratio of more than 10 standard deviations above the no amino donor control. By calculating these ratios we help control for variation in mass spectrometry desorption/ionization efficiency across the surface (27). The fractional conversion ratio was calculated by dividing the ion intensity for the conjugated transaminated amino donor by the ion intensity for the conjugated transaminated amino donor plus the ion intensity for conjugated keto acceptor. Historically, we have the author-used ratios of NIMS probe-labeled substrates and products, which is not possible for the oxime-MSI assay (because the oxime probe does not label amino donors). Therefore, an important goal for this initial analysis was to benchmark this approach for enzyme screening against previous reports because media conditions (*e.g.*, pH, ionic strength) may differentially impact the sensitivity of our assay for amino donors *versus* oxime-tagged molecules. Overall, the results from the oxime-MSI AT assays (Fig. 2*A*) had excellent correlation with those of previous biochemical characterization and showed that *At*TAT1 can use Tyr, histidine (His), leucine (Leu), methionine (Met), phenylalanine (Phe), and tryptophan (Trp) as amino donors in combination with ɑ-KG as a keto acceptor (15). Their keto acid products derived from these amino acid donor substrates were confidently detected after the oxime tagging and MSI (Fig. 2*B*). The Z-factor, a commonly used statistical measure of the quality of a high-throughput assay, ranged from 0.76 to 0.92 for all six hits, indicating an excellent assay and suitable for high-throughput screening (HTS) (32). To confirm that the oxime-MSI AT assay can be used in combination with other MSI techniques, activity of *At*TAT1 with Tyr with ɑ-KG as keto acceptor was analyzed with MALDI and yielded the same results as with NIMS (Fig. S2).

Given the potential of the oxime-MSI AT assay to determine amino acceptor preference, we also wanted to test our ability to quantify reaction yields. We expect that the mass spectrometry signal of an oxime-tagged analyte is proportional to all other oxime-tagged analytes, due to the mass spectrometry properties of the probe, and will rise monotonically with its concentration. This allows us to determine the relative preferences of amino donors and keto acceptors based on the ion counts in a semi-quantitative manner. This was confirmed by determining the relative activity of *At*TAT1 with Tyr and ɑ-KG, as lowering the Tyr concentrations resulted in lower fractional conversion ratios for the conversion of Tyr into 4-hydroxyphenylpyruvate (Fig. 2*C*).

Therefore, our new oxime-MSI AT assay using oxime tagging and subsequent MS analysis was able to accurately determine the substrate specificity of *At*TAT1.

### Characterization of *At*TAR1 against a panel of amino donors and keto acceptors

**Figure 3:**
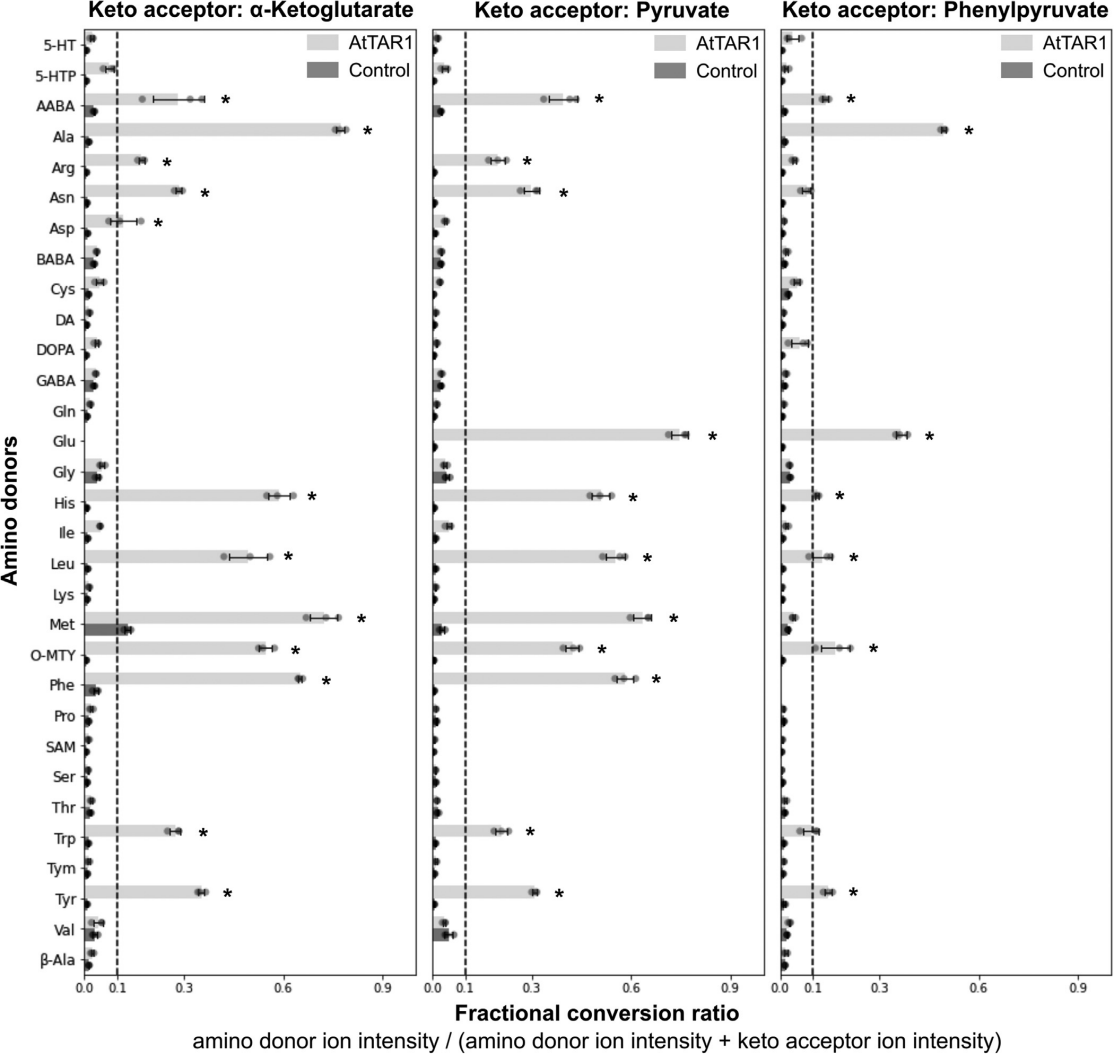
**Screening of *At*TAR1 against 31 amino donors and three keto acceptors.** Aminotransferase activity of *At*TAR1 was analyzed for 31 amino donors using three keto acceptors, ɑ-ketoglutarate, pyruvate, and phenylpyruvate. Bar chart shows the fractional conversion ratios for each amino donor with AtTAT1 or the control (no amino donor) ± the standard deviations for three biological replicates. Individual biological replicates are plotted as *closed circles*. *Dotted line* indicates a fractional conversion ratio of 0.1. ∗ indicates a fractional conversion ratio of >0.1 and greater than 10 standard deviations compared to the control. 5-HT, serotonin; 5-HTP, 5-hydroxy-L-tryptophan; AABA, 2-aminobutyric acid; Ala, alanine; Arg, arginine; Asn, asparagine; Asp, aspartic acid; *At*TAT1, *Arabidopis thaliana* tyrosine aminotransferase 1; *At*TAR1, *Arabidopsis thaliana* tryptophan aminotransferase related protein 1; BABA, β-Aminobutyric acid; Cys, cysteine; DA, dopamine; DOPA, L-DOPA; GABA, γ-Aminobutyric acid; Gln, glutamine; Glu, glutamic acid; Gly, glycine; His, histidine; Ile, isoleucine; Leu, leucine; Lys, lysine; Met, methionine; O-MTY, O-methyl-Tyrosine; Phe, phenylalanine; Pro, proline; SAM, S-Adenosyl methionine; Ser, serine; Thr, threonine; Trp, tryptophan; Tym, tyramine; Tyr, tyrosine; Val, valine; β-Ala, β-Alanine.

To further explore the high-throughput potential of the oxime-MSI AT assay, we screened *At*TAT1, *A. thaliana* tryptophan AT of Arabidopsis 1 (*At*TAA1), and A. thaliana tryptophan AT-related protein 1 (*At*TAR1) for activity against 31 different amino donors using three different keto acid acceptors in a 384-well format using NIMS. The amino donor preference of *At*TAA1 – a tryptophan AT – has previously been determined and hence included as another control enzyme (33). *At*TAR1 encodes a protein that is 69% identical to *At*TAA1 and is suggested to be a part of the IPA-mediated biosynthetic pathway of the plant hormone auxin (2, 34). The oxime-MSI AT assay used in this study identified that *At*TAR1 can use 13 different amino donors and all three keto acceptors (Fig. 3). *At*TAR1 used the amino acids alanine (Ala), arginine (Arg), asparagine (Asn), aspartic acid (Asp), glutamic acid (Glu), His, Leu, Met, Phe, Trp, and Tyr as well as the nonproteinogenic amino acids 2-aminobutyric acid (AABA), and O-methyl-tyrosine (O-MTY) as amino donors. The specificity of *At*TAR1 for Ala, Glu, Leu, Met, Phe, and Tyr as amino donors by *At*TAA1 was confirmed by the oxime-MSI AT assay (Fig. S3). The only exception was that Trp, a previously confirmed amino donor of *At*TAA1, was slightly below the activity threshold. Additionally, *At*TAA1 was able to use AABA, Asn, Glu, His, and O-MTY as its amino donors. In terms of their keto acid acceptor specificity, both *At*TAR1 and *At*TAA1, preferred pyruvate and ɑ-ketoglutarate over phenylpyruvate. Overall, these results demonstrate that the amino donor specificity and keto acceptor preferences of *At*TAR1 are similar to those of *At*TAA1, except that *At*TAR1 was also able to use Arg and Asp as amino donors. The oxime-MSI AT assay also confirmed previously known substrates of *At*TAT1 and further revealed its ability to use AABA and O-MTY as amino donors (Fig. S4).

**Figure 4:**
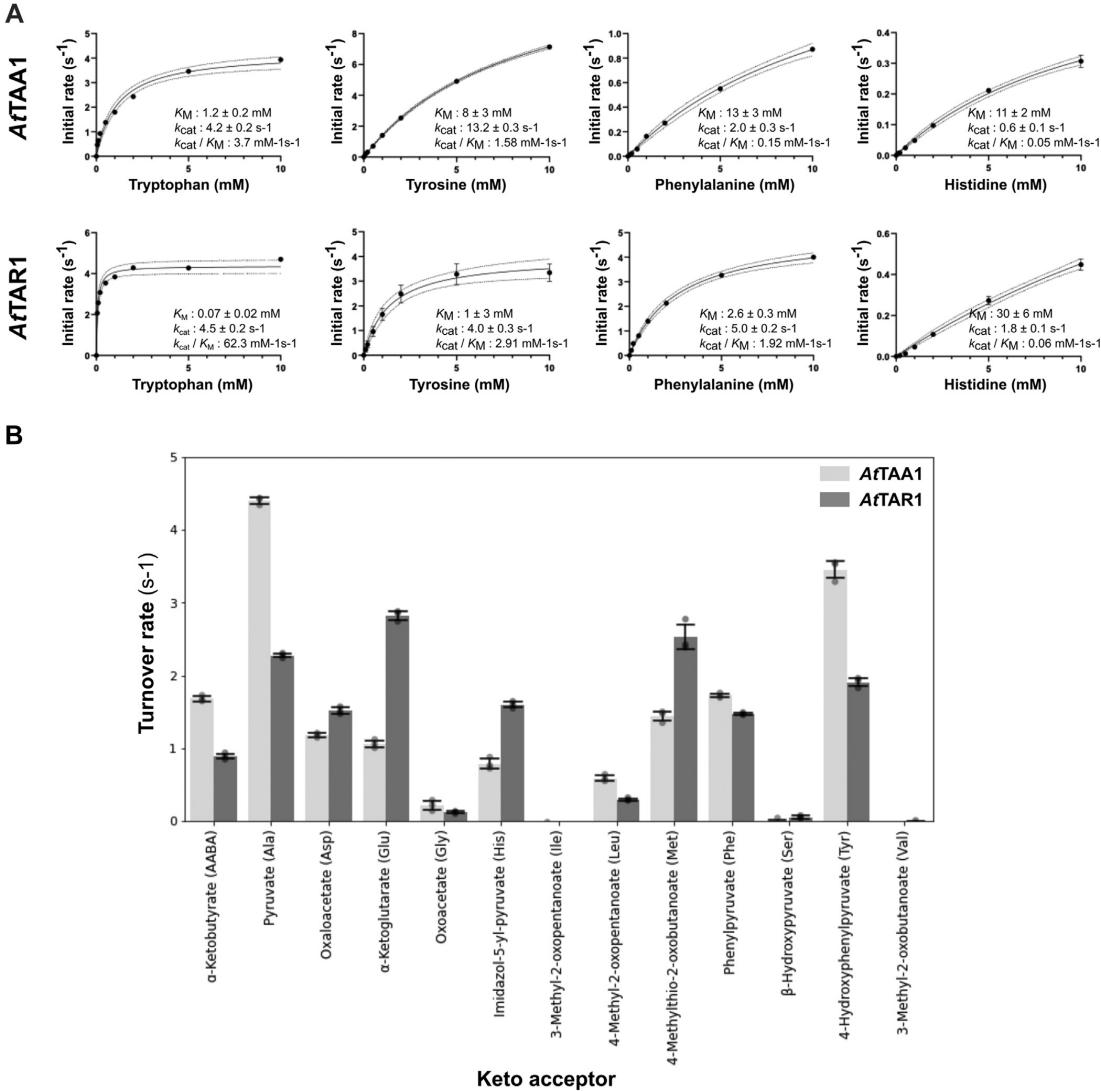
**Kinetic parameters and keto acid specificity screening of *At*TAA1 and *At*TAR1.***A*, kinetic properties of *At*TAA1 and *At*TAR1 with aromatic amino acid donors. *At*TAA1 and *At*TAR1 (1–10 ng/μl) were incubated with 20 mM keto acid (pyruvate or α-ketoglutarate) and varied concentrations of aromatic amino acids, when their activity increased linearly. Michaelis–Menten equation was fitted using non-linear regression for the calculation of kinetic parameters. Each data point is an average of three separate assays (n = 3). Error bars show SEM. *B*, tryptophan aminotransferase activity of *At*TAA1 and *At*TAR1 with 13 keto acceptors. X-axis shows the turnover rate of each enzyme with 40 mM tryptophan and 6 mM of keto acid given on the y-axis. Each bar is an average of three biological replicates (n = 3) and the error bar shows the standard deviation. Individual biological replicates are plotted as *closed circles*. Amino acids given in *parentheses* show the product formed by the transamination of the keto acid. AABA, 2-aminobutyric acid; Ala, Alanine; Asp, Aspartic acid; *At*TAA1, *Arabidopsis thaliana* tryptophan aminotransferase of *Arabidopsis* 1; *At*TAR1, *Arabidopsis thaliana* tryptophan aminotransferase related protein 1; Glu, glutamic acid; Gly, glycine; His, histidine; Ile, isoleucine; Leu, leucine; Met, methionine; Phe, phenylalanine; Ser, serine; Trp, tryptophan; Val, valine.

To validate the amino donor specificities identified by the oxime-MSI AT, kinetic parameters of *At*TAA1 and *At*TAR1 against aromatic amino acids (His, Phe, Trp, and Tyr) were determined using spectrophotometric assays that detects the formation of the analogues aromatic keto acids (Fig. 4*A*) (14). Both *At*TAA1 and *At*TAR1 had the highest catalytic efficiency (*k*_cat_/*K*_m_) with Trp, followed by Tyr, Phe, and His. *At*TAR1 had higher *k*_cat_/*K*_m_ with all substrates than *At*TAA1, mainly due to its lower *K*_m_ toward aromatic amino acids (Fig. 4*A*). Since AT-catalyzed reactions are typically reversible and with their amino donor and keto acceptor specificities expected to be similar, the keto acceptor specificity of *At*TAA1 and *At*TAR1 was analyzed to confirm oxime-MSI assay hits. Here, a spectrophotometric tryptophan AT assay using the Salkowski reagent was used to detect IPA generated from Trp by these two tryptophan ATs in the presence of various keto acceptor substrates (14, 17). This assay showed that both *At*TAA1 and *At*TAR1 can use pyruvate, ɑ-ketoglutarate, 4-methylthio-2-oxobutanoate, phenylpyruvate, imidazol-5-yl-pyruvate, 4-hydroxyphenylpyruvate, oxaloacetate, 4-methyl-2-oxopentanoate, and ɑ-ketobutyrate (Fig. 4*B*). These keto acid substrates correspond to Ala, Glu, Met, Phe, His, Tyr, Asp, Leu, and AABA, respectively, which were detected by the oxime-MSI AT assay in the reverse reactions (Fig. 3). Therefore, three independent assays, including enzyme kinetic analysis, showed consistent results and revealed that TAA1 and TAR1 have similar specificities to amino acids and their corresponding keto acids.

## Discussion

The oxime-MSI AT assay enables the detection of transaminated substrates from AT reactions in a high-throughput manner using a MS-based readout without the need of chromatographic separation. Similar to other AT assays, reactions can be carried out using native amino donors and keto acceptors but without the need of a coupled assay or relying on the intrinsic absorbance or fluorescence, which are specific to individual amino donors or keto acceptors (*e.g.*, those containing aromatic groups). As the detection relies on the tagging of the oxime probe to a ketone, the assay can detect all AT reactions that result in the formation of ketone from an amino donor, even beyond amino acids. Therefore, potential AT activity against target molecules relevant to the pharmaceutical and fine chemical industries, like valuable amine drug intermediates, can be screened (35, 36). Also, the oxime probe transfers favorable mass spectrometry properties to the reaction product, increasing the assay sensitivity. We assumed that the coupling efficiency of the oxime probe to all deaminated amino donors is equal as this was shown for other ketone/aldehyde substrates in a previous study (28). The only requirement for the transamination reaction is the formation of a stable ketone. If a reaction product is unstable or highly reactive and loses its ketone group, it cannot be derivatized. For example, the product of glutamine transamination is α-ketoglutaramate that reversibly cyclizes to a lactam (2-hydroxy-5-oxoproline), leaving only ∼0.3% present in the open-chain conformation at physiological pH (37). Only the open-chain form of α-ketoglutaramate can be tagged and thus detected.

Furthermore, we showed that the oxime-MSI AT assay can detect AT activity in a semi-quantitative manner allowing for relative quantification, as lowering of the Tyr concentration resulted in proportionally lower fractional conversion ratios of Tyr into hydroxyphenylpyruvate (Fig. 2*C*). For absolute quantification of the reaction, the use of an internal standard is required. This can be achieved by spiking in a ketone or aldehyde at a fixed concentration during oxime tagging, the ratio of the mass spectral intensity of tagged transaminated amino donor relative to the spiked in ketone/aldehyde can be used to determine the concentration of transaminated amino donor. The internal standard has to have a different mass than the expected transaminated amino donors or the keto acceptor and preferably has the same or a similar chemical structure (*e.g.*, stable isotope-labeled compounds) to minimize differences in tagging and desorption/ionization efficiencies. However, absolute quantification of selected AT activities, detected from the oxime AT screening, can be easily carried out by other conventional methods, which will provide additional validation, as we demonstrated here (Fig. 4).

We envision that the oxime-MSI AT assay will be used as an HTS platform to identify AT activity with enzyme, amino donor, and keto acceptor combinations, and that ‘hits’ are validated and characterized in depth by traditional AT activity assays, such as LC/MS or spectroscopy-based methods.

Besides NIMS, other MSI techniques can be used in combination with the oxime-MSI AT assay. The activity of *At*TAT1 with Tyr and ɑ-KG was analyzed using MALDI (Fig. S2). Although NIMS has its advantages over MALDI, including the lack of the requisite addition of matrix and crystal formation and the absence of matrix ions obscuring or interfering with analysis, we understand that MALDI is the most widely used MSI technique, and this shows that the oxime-MSI AT assay is compatible with MALDI analysis (26).

For determining AT activity, we set an arbitrary threshold for fractional conversion rates at >0.1 and it had to be more than 10 standard deviations above the no amino donor control. A fractional conversion rate of 0.1 indicates that the tagged AT reaction product had at least a signal intensity of 10% compared to all oxime-tagged reaction products and keto acceptor. This ensures that the peak of the tagged reaction product ion has a good signal-to-noise ratio. By setting the threshold of 10 standard deviations above the no amino donor control, it ensures that the reaction product was the result of transamination and not background activity or contamination. If the applied threshold is too high, it could produce false negatives. However, lowering the threshold to >0.05 only yields activity with four additional amino donors in combination with all three keto acids and the three ATs, indicating that the threshold is not too strict. For amino acids whose keto acids are commercially available, we confirmed the activities of both *At*TAA1 and *At*TAR1 observed in the oxime-MSI AT assay using a spectrophotometric-based AT assay, indicating that no false positives were observed.

HTS is achieved using acoustic sample deposition and the subsequent analysis by MSI. Here, we arrayed reactions onto a NIMS surface, but other desorption-based MS techniques can be used, including nanospray desorption electrospray ionization and desorption/ionization on silicon (26, 38). Up to 10,000 samples can be spotted onto a NIMS surface and can be analyzed in about 13 h, yielding a sample throughput of 5 s per sample (38). This is a far greater throughput than conventional separation–based MS platforms, such as LC/MS (38). Furthermore, acoustic sample deposition allows for using low reaction volumes and multi-well microplates which reduces costs and makes the assay amenable for automated liquid handling. Overall, the oxime-MSI AT assay can operate at the scale and throughput that is needed to investigate the large number of AT gene candidates and fully characterize the broad substrate specificity/promiscuity of ATs.

The oxime-MSI AT assay carried out in this study confirmed that *At*TAR1 has a similar amino donor specificity and keto acceptor preferences as *At*TAA1 (Figs. 3 and S3). This is not surprising, as TAR1 and TAA1 are phylogenetically related proteins, and prior genetic studies suggested that both function in the auxin biosynthetic pathway *via* indole-3-pyruvic acid (34, 39). Both ATs had a preference for ɑ-ketoglutarate and pyruvate as keto acceptors (Figs. 3, 4 and S3). *At*TAR1 was able to use more diverse amino donors than *At*TAA1---13 vs 11 amino donors, respectively. The exception is Asp, which was only used by *At*TAR1 as an amino donor in combination with ɑ-ketoglutarate as a keto acceptor. Both *At*TAT1 and *At*TAR1 can use Trp as an amino donor and showed activity above the initial threshold with Trp (Figs. 3 and S4). Lowering of the detection threshold would also confirm the usage of Trp by *At*TAA1 (Fig. S3), a known amino donor for *At*TAA1 (33). A prior study showed that TAA1 is feedback-inhibited by the transaminated product of Trp, indole-3-pyruvate, which could explain the low fractional conversion rate for Trp by TAA1 and not for TAT1 or TAR1 (40).

Enzyme kinetic analysis further validated that *At*TAA1 and *At*TAR1 are *bona fide* tryptophan ATs, since both enzymes showed significantly higher catalytic efficiency with Trp compared to other aromatic amino acids (Fig. 4*A*). Interestingly, however, *At*TAR1 had an ∼17 fold higher catalytic efficiency than *At*TAA1, mainly because of its much lower *K*_m_ with Trp, suggesting that TAR1 can function at much lower Trp concentrations than *At*TAA1 *in planta*. AABA and O-MTY were identified as novel substrates for all three ATs (Figs. 3, S3 and S4). AABA has a similar structure to that as Ala, and other ATs that were able to use Leu, Ala, Met, and Phe are also able to use AABA as an amino donor (41). This finding also demonstrates that the easily scalable nature of the oxime MS assay enables detection of novel AT activity and also provides rich experimental data to facilitate structure–function analyses of the AT enzyme family.

Our analyses of the keto acid specificity by spectrophotometric assays showed similar substrate preferences between amino acids and their corresponding keto acids for *At*TAA1 and *At*TAR1 (Figs. 3 and 4). For example, Glu, Ala, Met, His, Tyr, Phe, and Leu act as amino donors, and their corresponding keto acids, ɑ-ketoglutarate, pyruvate, 4-methylthio-2-oxobutanoate, imidazol-5-yl-pyruvate, 4-hydroxyphenylpyruvate, phenylpyruvate, and 4-methyl-2-oxopentanoate act as keto acceptors. Therefore, the amino donor substrate specificity determined by the oxime-MSI assay for a certain AT will likely reflect its keto acid substrate specificity. In contrast to *At*TAA1 that clearly prefers pyruvate and 4-hydroxyphenylpyruvate as keto acceptors, *At*TAR1 showed a broader keto acid specificity with similar preferences to pyruvate, ɑ-ketoglutarate, 4-methylthio-2-oxobutanoate, 4-hydroxyphenylpyruvate, oxaloacetate, phenylpyruvate, imidazole-5-yl-pyruvate (Fig. 4). However, the physiological functions of *At*TAR1’s broad keto acid specificity remain to be investigated *in vivo*. Interestingly, the activity of *At*TAA1 and *At*TAR1 with pyruvate and ɑ-ketobutyrate showed a similar pattern; TAA1 showing roughly double the activity of *At*TAR1 with both substrates (Fig. 4). Considering pyruvate and ɑ-ketobutyrate are similar in their structure, substrate specificity screenings can potentially be used to predict AT activity with structurally similar compounds (33, 40).

In conclusion, here we established the oxime-MSI AT assay and demonstrated its suitability for HTS of AT amino donor and keto acceptor specificity. This new technology enabled screening of substrate specificity and revealed that *At*TAR1 can use 13 amino acids as donors. In future studies, these properties could be used to build enzyme structure–function models, where experimental data gathered on representative AT enzymes is used to predict substrate specificities of unknown ATs based on phylogenetic relatedness, AT sequence and structure, and chemical properties of the substrates. Thus, this novel technology unlocks an exciting opportunity to expand our knowledge of AT characteristics and functions in N metabolic networks through comprehensive determination of AT substrate specificity.

## Experimental procedures

All individual chemicals were purchased from Sigma Aldrich except for SAM (New England Biolabs). Water (Honeywell International Inc), acetonitrile (OmniSolv, Sigma Aldrich), and methanol (J.T.Baker) were of LC-MS grade.

### Cloning of plasmid(s) for recombinant expression of ATs

The same pET28a expression construct described in Wang *et al*. (2016) was used for *A. thaliana* TyrAT 1 (TAT1, At5g53970). The entire coding sequence of tryptophan AT of Arabidopsis 1 (TAA1, AT1G70560) and tryptophan AT-related 1 (TAR1, AT1G23320) were synthesized by Synbio Technologies and cloned into the pET28a vector (Novagen) between the NdeI and BamHI sites.

### Recombinant production of ATs

Chemical competent Rosetta-2 (DE3) *Escherichia coli* cells (Novagen) were transformed with the pET28a vector expressing TAT1, TAA1, or TAR1 and selected on LB agar with 50 μg/ml kanamycin. Colonies for each construct were picked, inoculated in 10 ml LB medium with 50 μg/ml kanamycin, and incubated overnight at 37 °C, 200 rpm. 10 milliliters of the culture was transferred to 500 ml of fresh LB medium and grown at 37 °C, 200 rpm until *A*600 reached ∼0.6, when the temperature was dropped to 22 °C and Isopropyl β-d-1-thiogalactopyranoside was added at 0.2 mM final concentration. After overnight incubation at 30 °C, 200 rpm, cells were harvested by centrifugation at 6000 rpm for 20 min at 4 °C. The pellet was either stored at −80 °C for later use, or resuspended in 10 ml of lysis buffer containing 50 mM sodium phosphate (pH 8.0), 300 mM NaCl, 25 μM PLP, and 0.25 mg/ml lysozyme (Sigma Aldrich). After disrupting cells by three freeze-thaw cycles and sonication, the soluble extract was obtained by centrifugation at 4 °C, 18,000*g* for 30 min. His-tagged recombinant proteins were purified using a nickel-conjugated HisTrap Fastflow crude column (Cytiva) with ÄKTA pure chromatography system (Cytiva). Purified proteins were desalted with Sephadex G-50 superfine resin (Cytiva) into 100 mM HEPES buffer (pH 7.5) containing 25 μM PLP and 10% glycerol. Recombinant proteins were separated by SDS-PAGE, and the gels were stained by Coomassie Blue, imaged by ChemiDoc (Bio-Rad Laboratories, Inc), and their purity was assessed by ImageJ (https://imagej.net/ij/index.html) (Fig. S1).

### AT amino donor screening

All amino donor substrates were initially dissolved in 0.25 N NaOH at 100 mM, as the solubility of Tyrin water is low (<2 mM) (42). High-throughput AT reactions were tested in a reaction mixture with a final concentration of 100 mM Hepes pH 7.4, 10 mM amino donor, 6 mM keto acceptor, 0.5 mM PLP, and 20 to 40 ng/μl enzyme (0.3–5.12 ng/μl enzyme for kinetic studies), with a final pH of 7.6 to 8.0, which is around the optimum of *At*TAT1 and is similar to other plant aromatic amino acid AT activities and enzymes (15). Reactions were performed in 384-well plates with a final reaction volume of 2.5 μl. The reaction mixtures were incubated at 30 °C for 60 min and directly followed by oxime tagging.

### AT activity analysis by oxime tagging and NIMS or MALDI mass spectrometry

Transaminated amino donor substrates and nondepleted keto acid acceptor substrates were analyzed using oxime bioconjugate chemistry and NIMS. The synthesis and the subsequent oxime derivatization reactions with the O-alkyloxyamine fluorous tag (*m/z* 793.2365) were carried out as reported previously (28). Enzymatic reactions were diluted 1:5 using H_2_O. A 1 μl aliquot of the diluted enzymatic reactions was transferred into a 384 well plate containing 6 μl of 100 mM glycine acetate (pH 1.3), 3 μl of ethanol, 1 μl of O-alkyloxyamine fluorous tag [10 mM in 1:1 (v/v) water:methanol], and 0.26 μl of aniline per well. The mixture was incubated at room temperature (RT) for 16 h before NIMS or MALDI analysis.

For NIMS analysis, oxime reactions were prepared for either acoustic sample deposition or manual spotting onto a NIMS substrate which was processed as described previously (24). For each sample, 1 μl of the oxime reaction mixture, 5 μl water, 2 μl methanol, and 0.02 μl formic acid were combined. For acoustic sample deposition, samples were printed onto the NIMS surface using an ATS-100 acoustic transfer system (BioSera) with a sample deposition volume of 10 nl. Samples were printed in clusters of three biological replicates, with the microarray spot pitch (center-to-center distance) set at 900 μm. For manual spotting, samples (0.5 μl) were manually spotted onto the NIMS surface with three biological replicates.

For MALDI analysis, the oxime reaction mixture was mixed 1:1 with the matrix α-cyano-4-hydroxycinnamic acid (10 mg/ml in MeOH + 0.5% FA). Samples (0.8 μl) were manually spotted onto a stainless steel MALDI target plate with three biological replicates.

MS-based imaging was performed using a 5800 MALDI TOF/TOF (AB Sciex) mass spectrometer with laser intensity of 3000 to 4200 over a mass range of 500 − 2000 Da. Each position accumulated 20 laser shots. The instrument was controlled using the MALDI-MSI 4800 Imaging Tool using a 75 μm step size. Average ion intensity of the conjugated transaminated amino donor and keto acceptors substrates were determined using the OpenMSI Arrayed Analysis Toolkit (OMAAT) software package (31). MSI data obtained in this study is available and browsable at OpenMSI (43).

### Enzyme kinetic assays

Enzyme kinetic assays of *At*TAA1 and *At*TAR1 were conducted as described previously (14). Briefly, the assays were carried out in a reaction mixture containing 100 mM HEPES pH 8.0, 20 mM keto acceptor pyruvate (for *At*TAA1 with Trp, Tyr, and Phe) or ɑ-ketoglutarate (for *At*TAA1 with His and *At*TAR1 with Trp, Tyr, Phe, and His), 0.2 mM PLP, 1 to 10 ng/μl enzyme, and 0 to 10 mM final concentrations of amino acid donors. The reactions were initiated by the addition of the amino acid donor to the reaction mixture and incubated at 30 °C for five or 10 min, depending on the enzymatic rate, to achieve conditions where the product formation linearly increased with time and enzyme concentrations. Reactions with Trp, Tyr, and Phe were terminated by adding sodium hydroxide (final 0.4 M), and the reaction with His was terminated by boiling the reaction mixture for 10 min. Reactions with His were terminated by adding 1× volume of 1 M borate buffer pH 8.5, and cooled down to RT. Formation of phenylpyruvate, 4-hydroxyphenylpyruvate, indole-3-pyruvate, and imidazol-5-yl-pyruvate were detected by measuring the absorbance at 320, 331, 334, and 293 nm, respectively. Absorbance values were converted to concentration using standard curves of the corresponding keto acids. Kinetic parameters were calculated from the average of three separate assays by fitting the Michaelis–Menten equation using nonlinear regression function of GraphPad (https://www.graphpad.com/scientific-software/prism/).

### AT keto acceptor screening

Reverse reactions of tryptophan AT, *i.e.*, production of amino acids, were tested in a reaction mixture with a final concentration of 100 mM phosphate buffer pH 8.0, 40 mM tryptophan, 0.025 mM PLP, 1 ng/μl enzyme, and 6 mM of the keto acceptor given on the figure, in 300 μl final reaction volume. Reactions were initiated by the addition of tryptophan to the remaining components. The reaction mixtures were incubated at 30 °C for 5 min and terminated by the addition of 2× volume of Salkowski reagent (10 mM FeCl_3_ and 35% [v/v] H_2_SO_4_). After incubating at RT for 10 min in the dark, the formation of the pink/purple product was measured spectrophotometrically at λ530 nm using the Infinite M Plex plate reader (Tecan Group Ltd). The reaction without any keto acid acceptor was used as the background control. Kinetic parameters were calculated with GraphPad. All enzyme assays were performed under the condition where the product formation increased proportionally to the enzyme concentration and the reaction time.

## Data availability

All MSI ion intensity data for the bioconjugates obtained using the OMAAT algorithm can be found in Table S1. The OMAAT Python code and Jupyter notebooks are available at GitHub: https://github.com/biorack/omaat.

Raw MSI data can be found and is browsable at OpenMSI: http://openmsi.nersc.gov. TAT NIMS MSI dataset:

20210330MdR_5800_NIMS_AT_screen_TAT.h5 (https://openmsi.nersc.gov/openmsi/client/viewer/?file=%2Fproject%2Fprojectdirs%2Fopenmsi%2Fomsi_data_private%2Fraad0102%2F20210330MdR_5800_NIMS_AT_screen_TAT.h5&dataIndex=0&expIndex=0&channel1Value=905.3&channel1RangeValue=0.05&channel2Value=978.3&channel2RangeValue=0.05&channel3Value=929.3&channel3RangeValue=0.05&rangeValue=0.05&cursorCol1=96&cursorRow1=64&cursorCol2=192&cursorRow2=128&enableClientCache=false);

TAA NIMS MSI dataset: 20210402MdR_5800_NIMS_AT_screen_TAA1.h5 (https://openmsi.nersc.gov/openmsi/client/viewer/?file=%2Fproject%2Fprojectdirs%2Fopenmsi%2Fomsi_data_private%2Fraad0102%2F20210402MdR_5800_NIMS_AT_screen_TAA1.h5&dataIndex=0&expIndex=0&channel1Value=905.3&channel1RangeValue=0.2&channel2Value=978.3&channel2RangeValue=0.13876672116&channel3Value=929.3&channel3RangeValue=0.13876672116&rangeValue=0.2&cursorCol1=96&cursorRow1=64&cursorCol2=192&cursorRow2=128&enableClientCache=false);

TAR NIMS MSI dataset: 20210401MdR_5800_NIMS_AT_screen_TAR1.h5 (https://openmsi.nersc.gov/openmsi/client/viewer/?file=%2Fproject%2Fprojectdirs%2Fopenmsi%2Fomsi_data_private%2Fraad0102%2F20210401MdR_5800_NIMS_AT_screen_TAR1.h5&dataIndex=0&expIndex=0&channel1Value=905.3&channel1RangeValue=0.2&channel2Value=978.3&channel2RangeValue=0.2&channel3Value=929.3&channel3RangeValue=0.25&rangeValue=0.2&cursorCol1=96&cursorRow1=64&cursorCol2=192&cursorRow2=128&enableClientCache=false).

## Supporting information

This article contains supporting information.

## Conflict of interest

The authors declare that they have no conflict of interest with the contents of this article.
