# Supplementary material for: Mass spectrometry imaging–based assays for aminotransferase activity reveal a broad substrate spectrum for a previously uncharacterized enzyme
Source: J Biol Chem. 2023 Jan 24;299(3):102939. doi: 10.1016/j.jbc.2023.102939 (PMC9957770; doi:10.1016/j.jbc.2023.102939)
Supplement: Supplemental Figure Legends [file mmc1.docx]

**Mass spectrometry imaging-based assays for aminotransferase activity reveal a broad substrate spectrum for a previously uncharacterized enzyme**

Markus de Raad, Kaan Koper, Kai Deng, Benjamin P. Bowen, Hiroshi A. Maeda, Trent R. Northen

**Supplemental figure legends**

**Supplementary Figure 1. Purification of aminotransferases.** The three different recombinant aminotransferases were expressed in *E.coli* Rosetta-2 (DE3) cells and purified using imidazole metal affinity chromatography and buffer exchanged into 100 mM HEPES buffer (pH 7.5) containing 25 µM PLP and 10% glycerol. Five and ten microliter of desalted elute was loaded onto a polyacrylamide gel with 7.5% stacking and 10% separating phases. Representative SDS-PAGE gels stained with Coomassie Blue are shown.

**Supplementary Figure 2. Screening of *At*TAT1 against tyrosine as amino donor and ɑ-ketoglutarate as keto acceptor using MALDI.**

AT activity of *At*TAT1 was analyzed by MALDI using tyrosine as amino donor and ɑ-ketoglutarate as keto acceptor. Bar chart shows the fractional conversion ratios for tyrosine with AtTAT1, the no enzyme control (no AtTAT1) or the no amino donor control (no Tyr) +/- the standard deviations for 3 biological replicates. Individual biological replicates are plotted as closed circles. The fractional conversion ratio was calculated by dividing the average ion intensity (peak height) for the conjugated transaminated amino donor by the average ion intensity for the conjugated transaminated amino donor plus the average ion intensity for conjugated keto acceptor. Dotted line indicates a fractional conversion ratio of 0.1. Tyr, tyrosine.

**Supplementary Figure 3.**  **Screening of *At*TAA1 against 31 amino donors and 3 keto acceptors**.

AT activity of *At*TAR1 was analyzed for 31 amino donor using 3 keto acceptors, ɑ-ketoglutarate, pyruvic acid and phenylpyruvic acid. Bar chart shows the fractional conversion ratios for each amino donor with AtTAT1 or the control (no amino donor) +/- the standard deviations for 3 biological replicates. Individual biological replicates are plotted as closed circles. The fractional conversion ratio was calculated by dividing the average ion intensity (peak height) for the conjugated transaminated amino donor by the average ion intensity for the conjugated transaminated amino donor plus the average ion intensity for conjugated keto acceptor. Dotted line indicates a fractional conversion ratio of 0.1. * indicates a fractional conversion ratio of >0.1 and greater than 10 standard deviations compared to the control.

5-HT, serotonin; 5-HTP, ​​5-hydroxy-L-tryptophan; AABA, 2-Aminobutyric acid; Ala, alanine; Arg, arginine; Asn, asparagine; Asp, aspartic acid; BABA, β-Aminobutyric acid; Cys, cysteine; DA, dopamine; DOPA, L-DOPA; GABA, γ-Aminobutyric acid; Gln, glutamine; Glu, glutamic acid; Gly, glycine; His, histidine; Ile, isoleucine; Leu, leucine; Lys, lysine; Met, methionine; O-MTY, O-methyl-L-tyrosine; Phe, phenylalanine; Pro, proline; SAM, S-Adenosyl methionine; Ser, serine; Thr, threonine; Trp, tryptophan; Tym, tyramine; Tyr, tyrosine; Val, valine; β-Ala, β-Alanine.

**Supplementary Figure 4.**  **Screening of *At*TAT1 against 31 amino donors and 3 keto acceptors**.

AT activity of *At*TAR1 was analyzed for 31 amino donor using 3 keto acceptors, ɑ-ketoglutarate, pyruvic acid and phenylpyruvic acid. Bar chart shows the fractional conversion ratios for each amino donor with AtTAT1 or the control (no amino donor) +/- the standard deviations for 3 biological replicates. Individual biological replicates are plotted as closed circles. The fractional conversion ratio was calculated by dividing the average ion intensity (peak height) for the conjugated transaminated amino donor by the average ion intensity for the conjugated transaminated amino donor plus the average ion intensity for conjugated keto acceptor. Dotted line indicates a fractional conversion ratio of 0.1. * indicates a fractional conversion ratio of >0.1 and greater than 10 standard deviations compared to the control.

5-HT, serotonin; 5-HTP, ​​5-hydroxy-L-tryptophan; AABA, 2-Aminobutyric acid; Ala, alanine; Arg, arginine; Asn, asparagine; Asp, aspartic acid; BABA, β-Aminobutyric acid; Cys, cysteine; DA, dopamine; DOPA, L-DOPA; GABA, γ-Aminobutyric acid; Gln, glutamine; Glu, glutamic acid; Gly, glycine; His, histidine; Ile, isoleucine; Leu, leucine; Lys, lysine; Met, methionine; O-MTY, O-methyl-L-tyrosine; Phe, phenylalanine; Pro, proline; SAM, S-Adenosyl methionine; Ser, serine; Thr, threonine; Trp, tryptophan; Tym, tyramine; Tyr, tyrosine; Val, valine; β-Ala, β-Alanine.
