# Supplementary figures and images for: Mass spectrometry imaging–based assays for aminotransferase activity reveal a broad substrate spectrum for a previously uncharacterized enzyme
Source: J Biol Chem. 2023 Jan 24;299(3):102939. doi: 10.1016/j.jbc.2023.102939 (PMC9957770; doi:10.1016/j.jbc.2023.102939)

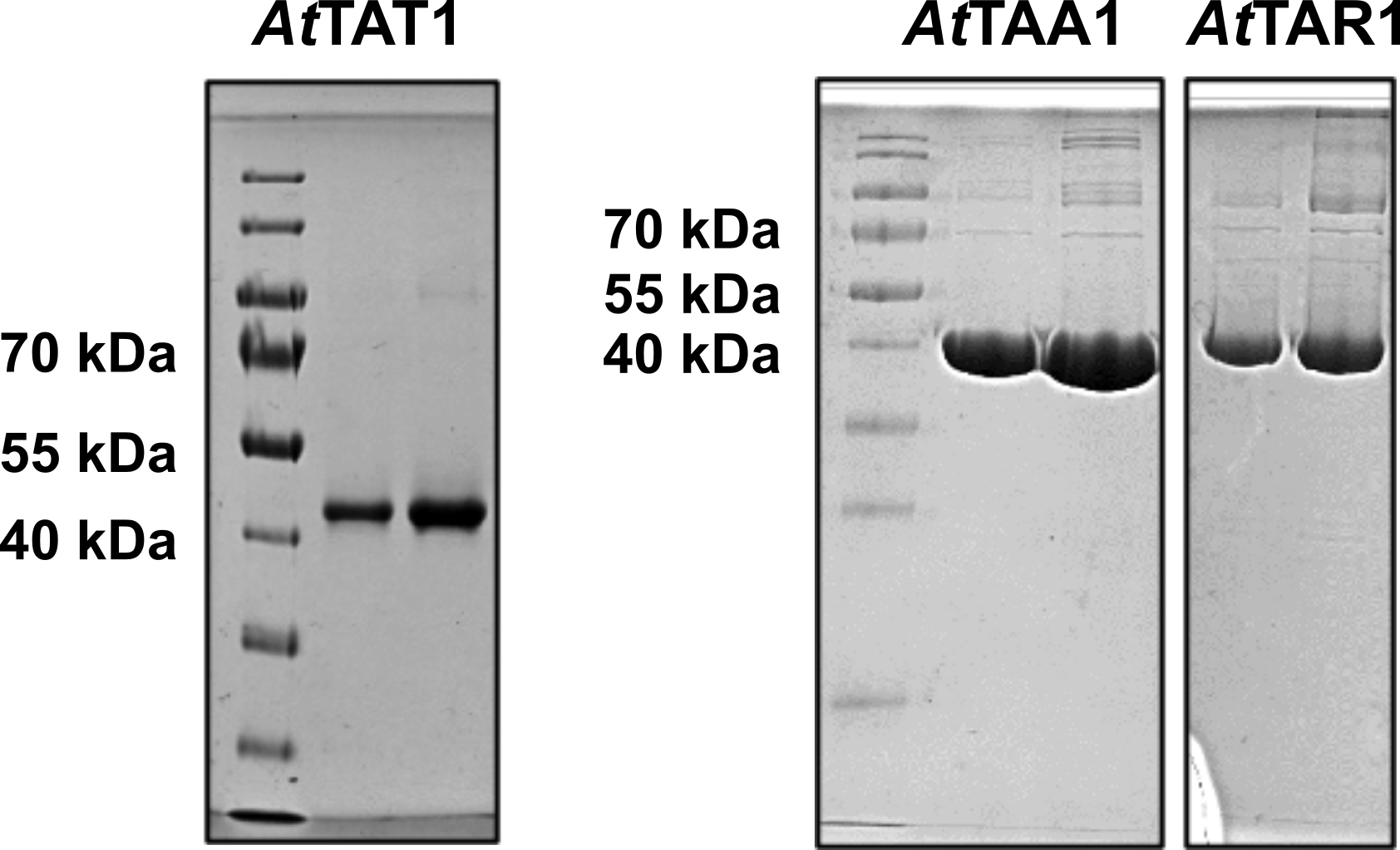

Supplement: Supplemental Figure S1 [file figs1.jpg]

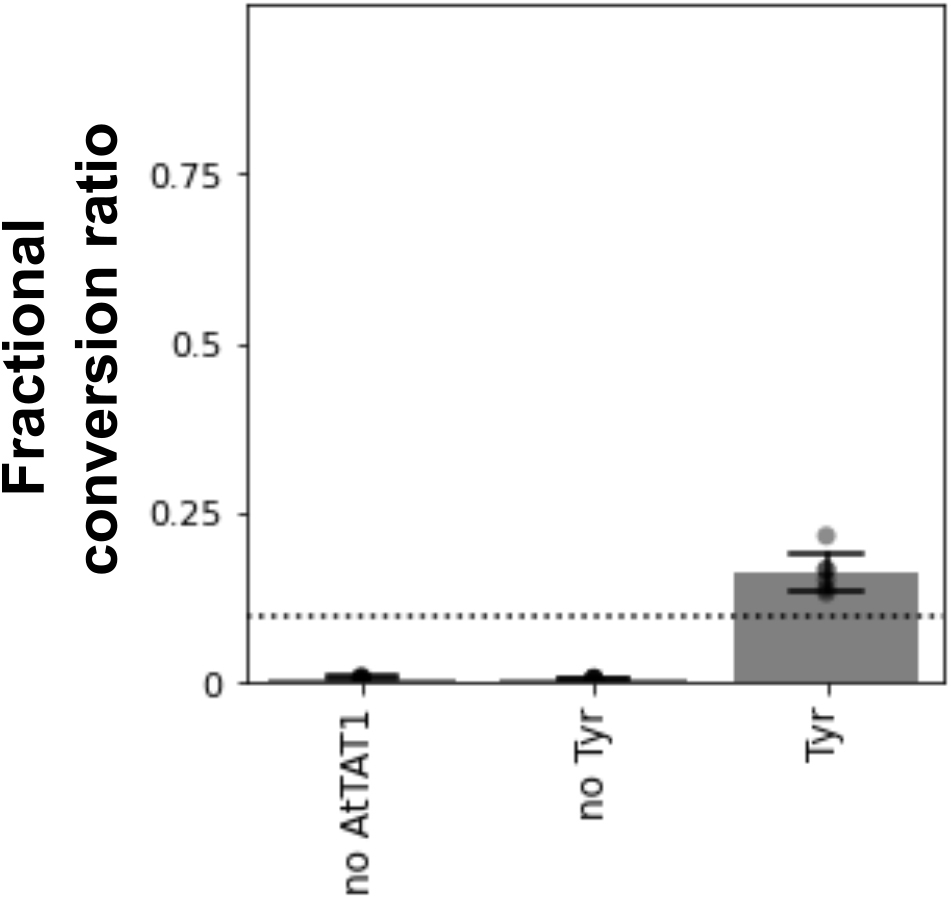

Supplement: Supplemental Figure S2 [file figs2.jpg]

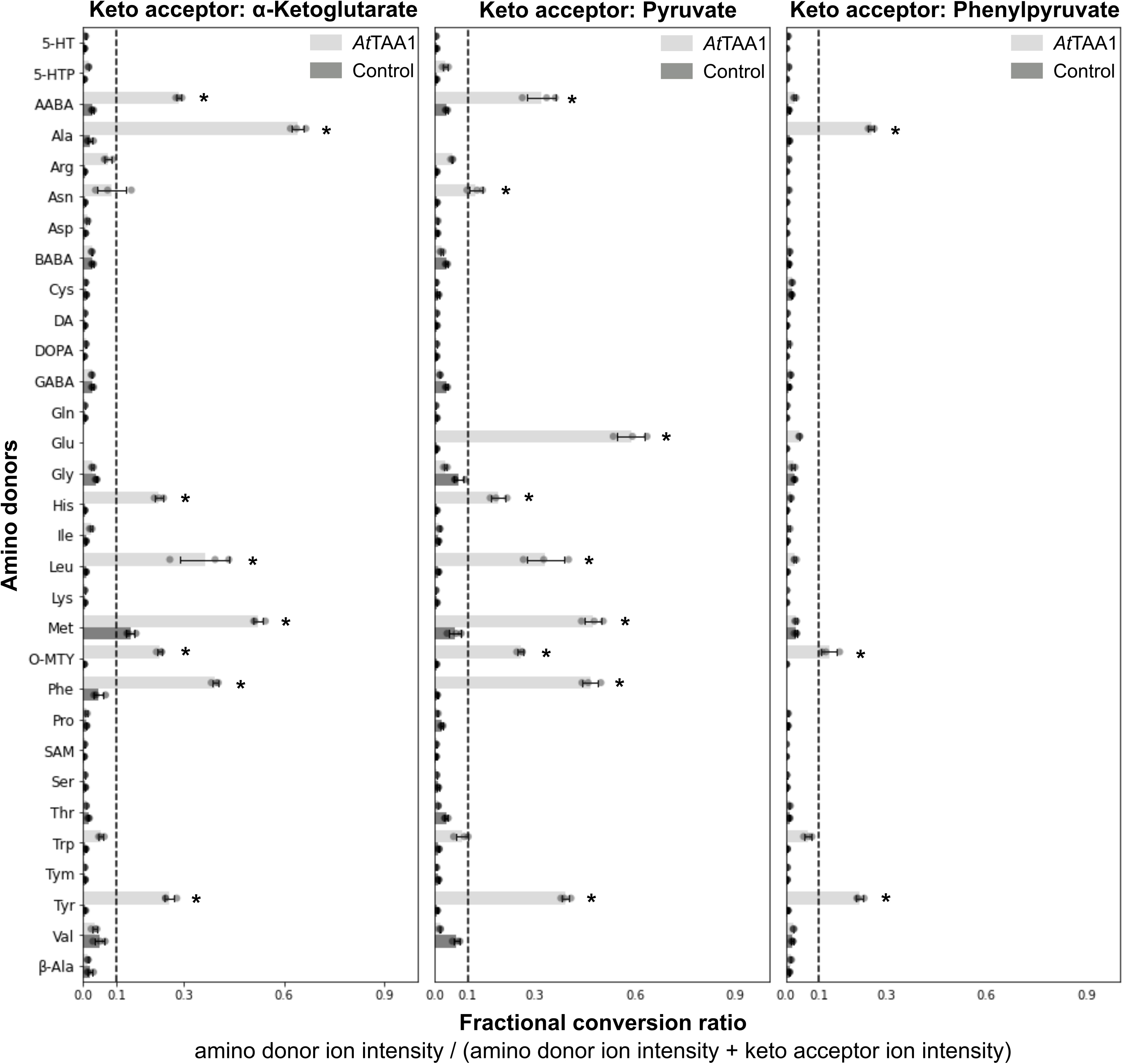

Supplement: Supplemental Figure S3 [file figs3.jpg]

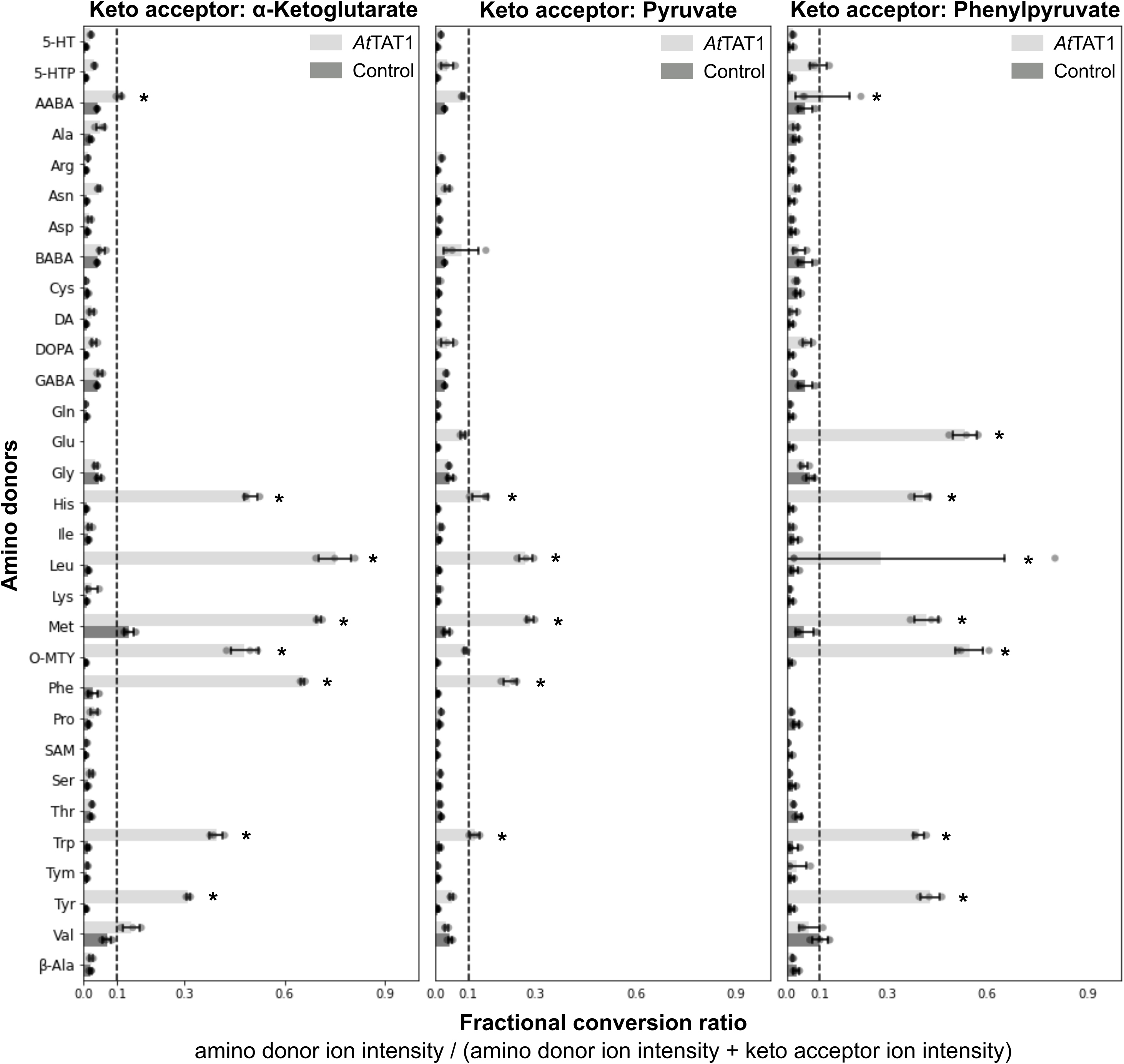

Supplement: Supplemental Figure S4 [file figs4.jpg]
